# (*Z*)-4-(2-Hy­droxy­benzyl­idene)-1-methyl-2-phenyl-1*H*-imidazol-5(4*H*)-one

**DOI:** 10.1107/S1600536812007921

**Published:** 2012-02-29

**Authors:** Ming-Jen Chang, Hsing-Yang Tsai, Tzu-Chien Fang, Ming-Hui Luo, Kew-Yu Chen

**Affiliations:** a Department of Chemical Engineering, Feng Chia University, 40724 Taichung, Taiwan

## Abstract

In the title compound, C_17_H_14_N_2_O_2_, the asymmetric unit comprises two mol­ecules that are comformationally similar [the dihedral angles between the phenyl rings in each are 46.35 (2) and 48.04 (3)°], with the conformation stabilized by intra­molecular O—H⋯N hydrogen bonds, which generate *S*(7) rings. In the crystal, inversion-related mol­ecules are linked by pairs of weak C—H⋯O hydrogen bonds, forming dimers with an *R*_2_^2^(16) graph-set motif. Weak inter-ring π–π stacking is observed in the structure, the shortest centroid-to-centroid distance being 3.7480 (13) Å.

## Related literature

For the spectroscopy and preparation of the title compound, see: Chuang *et al.* (2011 ▶). For the applications of proton-transfer dyes, see: Chen & Pang (2010 ▶); Gryko *et al.* (2010 ▶); Han *et al.* (2010 ▶); Helal *et al.* (2010 ▶); Ikeda *et al.* (2010 ▶); Ito *et al.* (2011 ▶); Lim *et al.* (2011 ▶); Lins *et al.* (2010 ▶); Maupin *et al.* (2011 ▶); Santos *et al.* (2011 ▶); Tang *et al.* (2011 ▶). For a related structure, see: Chen *et al.* (2007 ▶). For graph-set theory of hydrogen bonds, see: Bernstein *et al.* (1995 ▶).
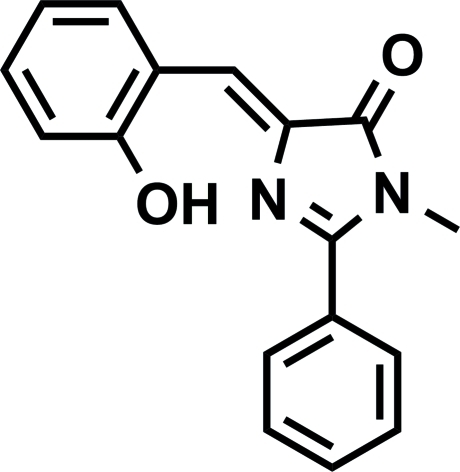


## Experimental

### 

#### Crystal data

C_17_H_14_N_2_O_2_
*M* *_r_* = 278.30
Triclinic, 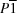

*a* = 9.7843 (4) Å
*b* = 9.8972 (3) Å
*c* = 15.4313 (6) Å
α = 72.086 (2)°
β = 79.301 (2)°
γ = 70.500 (2)°
*V* = 1334.57 (9) Å^3^
*Z* = 4
Mo *K*α radiation
μ = 0.09 mm^−1^
*T* = 150 K
0.32 × 0.28 × 0.14 mm

#### Data collection

Bruker SMART CCD diffractometer
Absorption correction: multi-scan (*SADABS*; Bruker, 2001 ▶) *T*_min_ = 0.889, *T*_max_ = 0.984
20392 measured reflections
4699 independent reflections
2273 reflections with *I* > 2σ(*I*)
*R*_int_ = 0.054

#### Refinement

*R*[*F* ^2^ > 2σ(*F* ^2^)] = 0.037
*wR*(*F* ^2^) = 0.101
*S* = 0.80
4699 reflections
382 parameters
H-atom parameters constrained
Δρ_max_ = 0.35 e Å^−3^
Δρ_min_ = −0.36 e Å^−3^

### 

Data collection: *SMART* (Bruker, 2001 ▶); cell refinement: *SAINT* (Bruker, 2001 ▶); data reduction: *SAINT*; program(s) used to solve structure: *SHELXS97* (Sheldrick, 2008 ▶); program(s) used to refine structure: *SHELXL97* (Sheldrick, 2008 ▶); molecular graphics: *ORTEP-3 for Windows* (Farrugia, 1997 ▶); software used to prepare material for publication: *WinGX* (Farrugia, 1999 ▶).

## Supplementary Material

Crystal structure: contains datablock(s) I, global. DOI: 10.1107/S1600536812007921/zs2183sup1.cif

Structure factors: contains datablock(s) I. DOI: 10.1107/S1600536812007921/zs2183Isup2.hkl

Supplementary material file. DOI: 10.1107/S1600536812007921/zs2183Isup3.cml

Additional supplementary materials:  crystallographic information; 3D view; checkCIF report

## Figures and Tables

**Table 1:** Hydrogen-bond geometry (Å, °)

*D*—H⋯*A*	*D*—H	H⋯*A*	*D*⋯*A*	*D*—H⋯*A*
O2—H2⋯N2	0.82	1.80	2.615 (2)	176
O4—H4⋯N4	0.82	1.79	2.612 (3)	175
C10—H10⋯O1^i^	0.93	2.58	3.403 (3)	148
C30—H30⋯O3^ii^	0.93	2.68	3.421 (3)	137

Symmetry codes: (i) 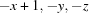; (ii) 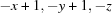.

**Fig. 1.**
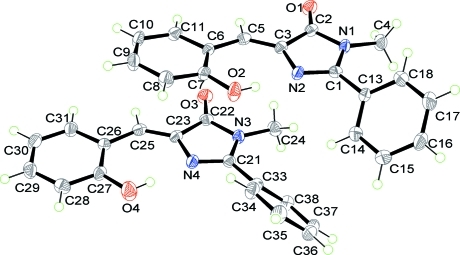
The molecular structure of the title compound, showing 50% probability displacement ellipsoids.

**Fig. 2.**
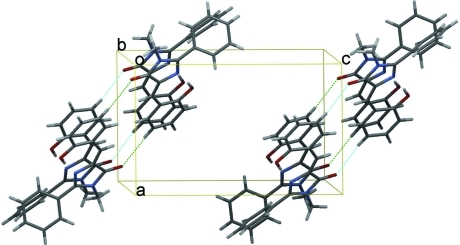
A section of the crystal packing of the title compound, viewed along the b axis. Green and blue dashed lines denote the intermolecular C10—H10···O1i and C30—H30···O3ii hydrogen bonds, respectively. For symmetry codes (i) and (ii), see Table 1.

## supplementary crystallographic information

### Comment

The excited-state intramolecular proton transfer (ESIPT) reaction of the title compound has been investigated recently (Chuang *et al.*, 2011), which involves transfer of a hydroxy proton to the imine nitrogen through an intramolecular seven-membered ring hydrogen-bonding system. The proton-transfer dyes have found many important applications. Prototypical examples are probes for solvation dynamics (Chen & Pang, 2010; Lins *et al.*) and biological environments (Lim *et al.*, 2011; Maupin *et al.*, 2011), near-infrared fluorescent dyes (Ikeda *et al.*, 2010), chemosensors (Han *et al.*, 2010; Helal *et al.*, 2010), photochromic materials (Ito *et al.*, 2011), fluorescence microscopy imaging (Santos *et al.*, 2011), and recent applications in the field of organic light-emitting devices (Gryko *et al.*, 2010; Tang *et al.*, 2011).

The molecular structure of the title compound, C_17_H_14_N_2_O_2_ is shown in Fig. 1. The asymmetric unit comprises two symmetry-independent molecules (*A* and *B*) which are comformationally similar [dihedral angles between the phenyl rings in each are 46.35 (2) and 48.04 (3)°]. The conformation stabilized in each by intramolecular O—H···N hydrogen bonds which generate *S*(7) rings (Chen *et al.*, 2007). Present also are intramolecular C—H···O interactions between the methyl group and the ketone O-atom, generating *S*(5) rings (Table 1). In the crystal (Fig. 2), inversion-related molecules are linked by pairs of weak hydrogen bonds (Table 1), forming cyclic dimers with an *R*_2_^2^(16) graph-set motif (Bernstein *et al.*, 1995). Weak π–π stacking is also observed in the crystal structure, the shortest centroid–centroid distance being 3.7480 (13) Å [symmetry code: *x*, *y* - 1, *z*].

### Experimental

The title compound was synthesized according to the literature procedure (Chuang *et al.*, 2011). Yellow needle-shaped crystals suitable for the crystallographic studies reported here were isolated over a period of six weeks by slow evaporation from a chloroform solution.

### Refinement

The C-bound H atoms were positioned geometrically (C—H = 0.93–0.96 Å) and allowed to ride on their parent atoms, with *U*_iso_(H) = 1.2*U*_eq_(C)]. The O-bound H atoms were positioned geometrically (O—H = 0.82 Å) and allowed to ride on their parent atoms, with *U*_iso_(H) = 1.5*U*_eq_(O)].

### Crystal data

**Table:**

C_17_H_14_N_2_O_2_	*Z* = 4
*M**_r_* = 278.30	*F*(000) = 584
Triclinic, *P*1	*D*_x_ = 1.385 Mg m^−^^3^
Hall symbol: -P 1	Mo *K*α radiation, λ = 0.71073 Å
*a* = 9.7843 (4) Å	Cell parameters from 3314 reflections
*b* = 9.8972 (3) Å	θ = 2.5–25.0°
*c* = 15.4313 (6) Å	µ = 0.09 mm^−^^1^
α = 72.086 (2)°	*T* = 150 K
β = 79.301 (2)°	Prism, yellow
γ = 70.500 (2)°	0.32 × 0.28 × 0.14 mm
*V* = 1334.57 (9) Å^3^	

### Data collection

**Table:**

Bruker SMART CCD diffractometer	4699 independent reflections
Radiation source: fine-focus sealed tube	2273 reflections with *I* > 2σ(*I*)
Graphite monochromator	*R*_int_ = 0.054
φ and ω scans	θ_max_ = 25.0°, θ_min_ = 1.4°
Absorption correction: multi-scan (*SADABS*; Bruker, 2001)	*h* = −11→11
*T*_min_ = 0.889, *T*_max_ = 0.984	*k* = −11→10
20392 measured reflections	*l* = −18→18

### Refinement

**Table:**

Refinement on *F*^2^	Secondary atom site location: difference Fourier map
Least-squares matrix: full	Hydrogen site location: inferred from neighbouring sites
*R*[*F*^2^ > 2σ(*F*^2^)] = 0.037	H-atom parameters constrained
*wR*(*F*^2^) = 0.101	*w* = 1/[σ^2^(*F*_o_^2^) + (0.049*P*)^2^] where *P* = (*F*_o_^2^ + 2*F*_c_^2^)/3
*S* = 0.80	(Δ/σ)_max_ < 0.001
4699 reflections	Δρ_max_ = 0.35 e Å^−^^3^
382 parameters	Δρ_min_ = −0.36 e Å^−^^3^
0 restraints	Extinction correction: *SHELXL97* (Sheldrick, 2008), Fc^*^=kFc[1+0.001xFc^2^λ^3^/sin(2θ)]^-1/4^
Primary atom site location: structure-invariant direct methods	Extinction coefficient: 0.037 (2)

### Special details

**Table:**

Geometry. All e.s.d.'s (except the e.s.d. in the dihedral angle between two l.s. planes) are estimated using the full covariance matrix. The cell e.s.d.'s are taken into account individually in the estimation of e.s.d.'s in distances, angles and torsion angles; correlations between e.s.d.'s in cell parameters are only used when they are defined by crystal symmetry. An approximate (isotropic) treatment of cell e.s.d.'s is used for estimating e.s.d.'s involving l.s. planes.
Refinement. Refinement of *F*^2^ against ALL reflections. The weighted *R*-factor *wR* and goodness of fit *S* are based on *F*^2^, conventional *R*-factors *R* are based on *F*, with *F* set to zero for negative *F*^2^. The threshold expression of *F*^2^ > σ(*F*^2^) is used only for calculating *R*-factors(gt) *etc*. and is not relevant to the choice of reflections for refinement. *R*-factors based on *F*^2^ are statistically about twice as large as those based on *F*, and *R*-factors based on ALL data will be even larger.

### Fractional atomic coordinates and isotropic or equivalent isotropic displacement parameters (Å^2^)

**Table:**

	*x*	*y*	*z*	*U*_iso_*/*U*_eq_	
O1	0.13687 (16)	−0.12970 (16)	0.02374 (11)	0.0292 (4)	
O2	0.24941 (16)	0.01412 (16)	0.32193 (9)	0.0331 (5)	
H2	0.1962	−0.0125	0.2996	0.050*	
O3	0.15388 (16)	0.37862 (16)	0.01672 (11)	0.0299 (4)	
O4	0.23819 (17)	0.52664 (17)	0.32188 (10)	0.0410 (5)	
H4	0.1961	0.4877	0.2999	0.061*	
N1	−0.00550 (19)	−0.16641 (18)	0.16225 (12)	0.0229 (5)	
N2	0.09021 (19)	−0.07230 (18)	0.24367 (12)	0.0219 (5)	
N3	0.00423 (19)	0.34215 (18)	0.15218 (12)	0.0228 (5)	
N4	0.10847 (19)	0.41218 (18)	0.24187 (12)	0.0227 (5)	
C1	−0.0086 (2)	−0.1355 (2)	0.24468 (15)	0.0223 (6)	
C2	0.1046 (2)	−0.1190 (2)	0.10228 (16)	0.0227 (6)	
C3	0.1655 (2)	−0.0577 (2)	0.15660 (15)	0.0206 (6)	
C4	−0.1125 (2)	−0.2116 (2)	0.13239 (15)	0.0306 (6)	
H4A	−0.1132	−0.1750	0.0671	0.046*	
H4B	−0.2073	−0.1713	0.1609	0.046*	
H4C	−0.0875	−0.3181	0.1495	0.046*	
C5	0.2778 (2)	−0.0006 (2)	0.12414 (15)	0.0215 (6)	
H5	0.3074	0.0033	0.0628	0.026*	
C6	0.3625 (2)	0.0560 (2)	0.16442 (15)	0.0193 (6)	
C7	0.3494 (2)	0.0583 (2)	0.25650 (16)	0.0232 (6)	
C8	0.4470 (3)	0.1070 (2)	0.28520 (16)	0.0307 (7)	
H8	0.4385	0.1081	0.3460	0.037*	
C9	0.5556 (3)	0.1535 (2)	0.22577 (16)	0.0312 (7)	
H9	0.6200	0.1846	0.2469	0.037*	
C10	0.5697 (2)	0.1544 (2)	0.13480 (16)	0.0288 (6)	
H10	0.6424	0.1868	0.0941	0.035*	
C11	0.4740 (2)	0.1065 (2)	0.10597 (15)	0.0251 (6)	
H11	0.4834	0.1074	0.0447	0.030*	
C13	−0.1095 (2)	−0.1721 (2)	0.32475 (15)	0.0213 (6)	
C14	−0.1679 (2)	−0.0741 (2)	0.37879 (15)	0.0259 (6)	
H14	−0.1459	0.0154	0.3627	0.031*	
C15	−0.2590 (2)	−0.1091 (2)	0.45685 (16)	0.0316 (7)	
H15	−0.2995	−0.0424	0.4925	0.038*	
C16	−0.2899 (3)	−0.2423 (3)	0.48168 (16)	0.0335 (7)	
H16	−0.3511	−0.2654	0.5342	0.040*	
C17	−0.2304 (3)	−0.3420 (3)	0.42909 (17)	0.0403 (7)	
H17	−0.2504	−0.4327	0.4465	0.048*	
C18	−0.1414 (3)	−0.3063 (2)	0.35063 (16)	0.0342 (7)	
H18	−0.1023	−0.3729	0.3147	0.041*	
C21	0.0052 (2)	0.3587 (2)	0.23784 (15)	0.0213 (6)	
C22	0.1193 (2)	0.3853 (2)	0.09563 (16)	0.0224 (6)	
C23	0.1837 (2)	0.4354 (2)	0.15464 (15)	0.0218 (6)	
C24	−0.0927 (2)	0.2851 (2)	0.12174 (15)	0.0300 (6)	
H24A	−0.0467	0.2512	0.0685	0.045*	
H24B	−0.1819	0.3626	0.1071	0.045*	
H24C	−0.1133	0.2037	0.1696	0.045*	
C25	0.2933 (2)	0.4973 (2)	0.12575 (15)	0.0241 (6)	
H25	0.3284	0.5009	0.0650	0.029*	
C26	0.3682 (2)	0.5595 (2)	0.16923 (16)	0.0219 (6)	
C27	0.3378 (2)	0.5747 (2)	0.25873 (16)	0.0238 (6)	
C28	0.4158 (3)	0.6439 (2)	0.28910 (16)	0.0288 (6)	
H28	0.3938	0.6553	0.3482	0.035*	
C29	0.5248 (3)	0.6955 (2)	0.23309 (17)	0.0314 (7)	
H29	0.5759	0.7410	0.2544	0.038*	
C30	0.5579 (2)	0.6795 (2)	0.14511 (17)	0.0319 (7)	
H30	0.6319	0.7136	0.1071	0.038*	
C31	0.4811 (2)	0.6132 (2)	0.11422 (16)	0.0289 (6)	
H31	0.5042	0.6032	0.0548	0.035*	
C33	−0.0964 (2)	0.3198 (2)	0.31732 (15)	0.0221 (6)	
C34	−0.0444 (3)	0.2553 (2)	0.40301 (16)	0.0295 (6)	
H34	0.0537	0.2357	0.4088	0.035*	
C35	−0.1373 (3)	0.2200 (2)	0.47980 (16)	0.0334 (7)	
H35	−0.1012	0.1757	0.5368	0.040*	
C36	−0.2833 (3)	0.2502 (2)	0.47222 (16)	0.0290 (6)	
H36	−0.3458	0.2261	0.5239	0.035*	
C37	−0.3360 (3)	0.3161 (2)	0.38798 (16)	0.0282 (6)	
H37	−0.4348	0.3376	0.3830	0.034*	
C38	−0.2438 (2)	0.3510 (2)	0.31025 (15)	0.0254 (6)	
H38	−0.2807	0.3952	0.2535	0.030*	

### Atomic displacement parameters (Å^2^)

**Table:**

	*U*^11^	*U*^22^	*U*^33^	*U*^12^	*U*^13^	*U*^23^
O1	0.0265 (10)	0.0404 (10)	0.0249 (11)	−0.0125 (8)	0.0015 (8)	−0.0138 (9)
O2	0.0353 (11)	0.0523 (11)	0.0223 (10)	−0.0289 (9)	0.0055 (9)	−0.0123 (8)
O3	0.0312 (11)	0.0353 (10)	0.0240 (11)	−0.0109 (9)	0.0004 (9)	−0.0096 (9)
O4	0.0483 (12)	0.0557 (12)	0.0318 (11)	−0.0307 (10)	0.0043 (10)	−0.0175 (9)
N1	0.0195 (12)	0.0289 (12)	0.0253 (13)	−0.0123 (10)	0.0000 (10)	−0.0099 (10)
N2	0.0184 (12)	0.0245 (11)	0.0230 (13)	−0.0092 (10)	0.0017 (10)	−0.0055 (9)
N3	0.0228 (13)	0.0275 (12)	0.0217 (12)	−0.0112 (10)	−0.0014 (10)	−0.0079 (10)
N4	0.0206 (12)	0.0259 (11)	0.0228 (13)	−0.0098 (10)	0.0011 (10)	−0.0068 (9)
C1	0.0199 (15)	0.0205 (14)	0.0256 (16)	−0.0033 (12)	−0.0017 (12)	−0.0078 (12)
C2	0.0187 (15)	0.0232 (14)	0.0241 (16)	−0.0041 (12)	−0.0015 (13)	−0.0061 (12)
C3	0.0170 (14)	0.0212 (13)	0.0217 (15)	−0.0042 (11)	−0.0019 (12)	−0.0044 (11)
C4	0.0265 (16)	0.0392 (16)	0.0340 (17)	−0.0156 (13)	−0.0043 (13)	−0.0136 (13)
C5	0.0208 (15)	0.0233 (14)	0.0171 (14)	−0.0032 (12)	−0.0008 (11)	−0.0049 (11)
C6	0.0164 (14)	0.0186 (13)	0.0208 (15)	−0.0044 (11)	−0.0010 (12)	−0.0036 (11)
C7	0.0217 (15)	0.0229 (14)	0.0244 (16)	−0.0086 (12)	0.0017 (12)	−0.0054 (12)
C8	0.0332 (16)	0.0412 (16)	0.0242 (16)	−0.0164 (14)	−0.0004 (13)	−0.0133 (13)
C9	0.0283 (16)	0.0406 (16)	0.0325 (17)	−0.0189 (13)	−0.0029 (13)	−0.0113 (13)
C10	0.0197 (15)	0.0357 (15)	0.0334 (17)	−0.0145 (13)	0.0025 (13)	−0.0084 (13)
C11	0.0227 (15)	0.0282 (14)	0.0239 (15)	−0.0070 (12)	0.0010 (12)	−0.0084 (12)
C13	0.0176 (14)	0.0243 (14)	0.0231 (15)	−0.0084 (12)	−0.0009 (12)	−0.0058 (12)
C14	0.0264 (15)	0.0258 (14)	0.0269 (15)	−0.0106 (12)	−0.0003 (12)	−0.0068 (12)
C15	0.0316 (16)	0.0329 (16)	0.0312 (17)	−0.0101 (13)	0.0045 (13)	−0.0135 (13)
C16	0.0296 (17)	0.0405 (16)	0.0319 (17)	−0.0190 (14)	0.0078 (13)	−0.0086 (14)
C17	0.0440 (18)	0.0402 (17)	0.0427 (18)	−0.0269 (15)	0.0126 (15)	−0.0134 (14)
C18	0.0376 (17)	0.0318 (16)	0.0386 (17)	−0.0162 (13)	0.0071 (14)	−0.0166 (13)
C21	0.0220 (15)	0.0184 (14)	0.0222 (16)	−0.0052 (12)	−0.0017 (12)	−0.0048 (12)
C22	0.0222 (15)	0.0193 (14)	0.0215 (16)	−0.0027 (11)	−0.0011 (13)	−0.0038 (12)
C23	0.0192 (14)	0.0233 (14)	0.0210 (15)	−0.0058 (12)	0.0015 (12)	−0.0057 (12)
C24	0.0318 (16)	0.0322 (15)	0.0303 (16)	−0.0111 (13)	−0.0054 (13)	−0.0109 (12)
C25	0.0229 (15)	0.0230 (14)	0.0227 (15)	−0.0039 (12)	0.0026 (12)	−0.0068 (12)
C26	0.0202 (15)	0.0194 (14)	0.0256 (16)	−0.0047 (12)	−0.0013 (12)	−0.0073 (12)
C27	0.0217 (15)	0.0236 (14)	0.0243 (16)	−0.0090 (12)	0.0012 (12)	−0.0032 (12)
C28	0.0324 (16)	0.0286 (14)	0.0257 (16)	−0.0059 (13)	−0.0073 (13)	−0.0081 (12)
C29	0.0250 (16)	0.0274 (15)	0.0443 (19)	−0.0077 (13)	−0.0107 (14)	−0.0087 (14)
C30	0.0205 (15)	0.0297 (15)	0.0430 (19)	−0.0080 (12)	0.0009 (13)	−0.0080 (14)
C31	0.0249 (16)	0.0252 (14)	0.0363 (17)	−0.0089 (12)	−0.0019 (13)	−0.0066 (13)
C33	0.0229 (15)	0.0215 (14)	0.0234 (16)	−0.0087 (12)	−0.0009 (12)	−0.0068 (12)
C34	0.0245 (15)	0.0384 (16)	0.0283 (16)	−0.0130 (13)	−0.0031 (13)	−0.0080 (13)
C35	0.0361 (17)	0.0436 (17)	0.0226 (16)	−0.0178 (14)	−0.0025 (13)	−0.0050 (13)
C36	0.0299 (17)	0.0327 (15)	0.0253 (16)	−0.0139 (13)	0.0061 (13)	−0.0091 (13)
C37	0.0207 (15)	0.0283 (15)	0.0354 (17)	−0.0094 (12)	0.0015 (13)	−0.0079 (13)
C38	0.0248 (15)	0.0257 (14)	0.0248 (15)	−0.0080 (12)	−0.0035 (12)	−0.0043 (12)

### Geometric parameters (Å, º)

**Table:**

O1—C2	1.222 (2)	C14—H14	0.9300
O2—C7	1.347 (2)	C15—C16	1.373 (3)
O2—H2	0.8200	C15—H15	0.9300
O3—C22	1.218 (2)	C16—C17	1.380 (3)
O4—C27	1.341 (2)	C16—H16	0.9300
O4—H4	0.8200	C17—C18	1.378 (3)
N1—C2	1.392 (3)	C17—H17	0.9300
N1—C1	1.390 (3)	C18—H18	0.9300
N1—C4	1.458 (2)	C21—C33	1.467 (3)
N2—C1	1.311 (2)	C22—C23	1.474 (3)
N2—C3	1.397 (3)	C23—C25	1.348 (3)
N3—C21	1.383 (3)	C24—H24A	0.9600
N3—C22	1.392 (3)	C24—H24B	0.9600
N3—C24	1.458 (2)	C24—H24C	0.9600
N4—C21	1.306 (2)	C25—C26	1.450 (3)
N4—C23	1.400 (3)	C25—H25	0.9300
C1—C13	1.464 (3)	C26—C27	1.402 (3)
C2—C3	1.471 (3)	C26—C31	1.410 (3)
C3—C5	1.347 (3)	C27—C28	1.396 (3)
C4—H4A	0.9600	C28—C29	1.376 (3)
C4—H4B	0.9600	C28—H28	0.9300
C4—H4C	0.9600	C29—C30	1.381 (3)
C5—C6	1.449 (3)	C29—H29	0.9300
C5—H5	0.9300	C30—C31	1.371 (3)
C6—C11	1.406 (3)	C30—H30	0.9300
C6—C7	1.409 (3)	C31—H31	0.9300
C7—C8	1.392 (3)	C33—C34	1.388 (3)
C8—C9	1.374 (3)	C33—C38	1.389 (3)
C8—H8	0.9300	C34—C35	1.382 (3)
C9—C10	1.382 (3)	C34—H34	0.9300
C9—H9	0.9300	C35—C36	1.378 (3)
C10—C11	1.372 (3)	C35—H35	0.9300
C10—H10	0.9300	C36—C37	1.373 (3)
C11—H11	0.9300	C36—H36	0.9300
C13—C14	1.383 (3)	C37—C38	1.387 (3)
C13—C18	1.388 (3)	C37—H37	0.9300
C14—C15	1.385 (3)	C38—H38	0.9300
			
C7—O2—H2	109.5	C18—C17—H17	120.2
C27—O4—H4	109.5	C16—C17—H17	120.2
C2—N1—C1	108.23 (18)	C17—C18—C13	120.6 (2)
C2—N1—C4	122.40 (19)	C17—C18—H18	119.7
C1—N1—C4	128.31 (19)	C13—C18—H18	119.7
C1—N2—C3	106.55 (18)	N4—C21—N3	112.87 (19)
C21—N3—C22	108.32 (18)	N4—C21—C33	122.4 (2)
C21—N3—C24	128.78 (18)	N3—C21—C33	124.7 (2)
C22—N3—C24	122.86 (19)	O3—C22—N3	125.6 (2)
C21—N4—C23	106.86 (19)	O3—C22—C23	130.9 (2)
N2—C1—N1	112.80 (19)	N3—C22—C23	103.46 (19)
N2—C1—C13	123.5 (2)	C25—C23—N4	127.5 (2)
N1—C1—C13	123.68 (19)	C25—C23—C22	124.1 (2)
O1—C2—N1	125.2 (2)	N4—C23—C22	108.41 (18)
O1—C2—C3	131.3 (2)	N3—C24—H24A	109.5
N1—C2—C3	103.43 (19)	N3—C24—H24B	109.5
C5—C3—N2	128.0 (2)	H24A—C24—H24B	109.5
C5—C3—C2	123.0 (2)	N3—C24—H24C	109.5
N2—C3—C2	108.99 (18)	H24A—C24—H24C	109.5
N1—C4—H4A	109.5	H24B—C24—H24C	109.5
N1—C4—H4B	109.5	C23—C25—C26	133.6 (2)
H4A—C4—H4B	109.5	C23—C25—H25	113.2
N1—C4—H4C	109.5	C26—C25—H25	113.2
H4A—C4—H4C	109.5	C27—C26—C31	117.1 (2)
H4B—C4—H4C	109.5	C27—C26—C25	126.9 (2)
C3—C5—C6	133.7 (2)	C31—C26—C25	115.9 (2)
C3—C5—H5	113.2	O4—C27—C28	114.2 (2)
C6—C5—H5	113.2	O4—C27—C26	125.9 (2)
C11—C6—C7	117.1 (2)	C28—C27—C26	120.0 (2)
C11—C6—C5	116.0 (2)	C29—C28—C27	121.1 (2)
C7—C6—C5	126.9 (2)	C29—C28—H28	119.4
O2—C7—C8	115.5 (2)	C27—C28—H28	119.4
O2—C7—C6	125.2 (2)	C28—C29—C30	119.8 (2)
C8—C7—C6	119.3 (2)	C28—C29—H29	120.1
C9—C8—C7	121.5 (2)	C30—C29—H29	120.1
C9—C8—H8	119.2	C31—C30—C29	119.5 (2)
C7—C8—H8	119.2	C31—C30—H30	120.2
C8—C9—C10	120.4 (2)	C29—C30—H30	120.2
C8—C9—H9	119.8	C30—C31—C26	122.4 (2)
C10—C9—H9	119.8	C30—C31—H31	118.8
C11—C10—C9	118.4 (2)	C26—C31—H31	118.8
C11—C10—H10	120.8	C34—C33—C38	118.9 (2)
C9—C10—H10	120.8	C34—C33—C21	118.9 (2)
C10—C11—C6	123.3 (2)	C38—C33—C21	122.2 (2)
C10—C11—H11	118.4	C35—C34—C33	120.6 (2)
C6—C11—H11	118.4	C35—C34—H34	119.7
C14—C13—C18	119.2 (2)	C33—C34—H34	119.7
C14—C13—C1	119.10 (19)	C36—C35—C34	120.2 (2)
C18—C13—C1	121.6 (2)	C36—C35—H35	119.9
C13—C14—C15	120.1 (2)	C34—C35—H35	119.9
C13—C14—H14	119.9	C37—C36—C35	119.6 (2)
C15—C14—H14	119.9	C37—C36—H36	120.2
C16—C15—C14	120.1 (2)	C35—C36—H36	120.2
C16—C15—H15	119.9	C36—C37—C38	120.8 (2)
C14—C15—H15	119.9	C36—C37—H37	119.6
C15—C16—C17	120.3 (2)	C38—C37—H37	119.6
C15—C16—H16	119.8	C33—C38—C37	119.9 (2)
C17—C16—H16	119.8	C33—C38—H38	120.1
C18—C17—C16	119.6 (2)	C37—C38—H38	120.1
			
C2—N1—C1—N2	−0.1 (2)	C3—C5—C6—C7	3.3 (4)
C2—N1—C1—C13	−179.03 (19)	C3—C5—C6—C11	179.8 (2)
C4—N1—C1—N2	−168.39 (19)	C5—C6—C7—O2	−3.5 (3)
C1—N1—C2—O1	179.9 (2)	C5—C6—C7—C8	175.3 (2)
C1—N1—C2—C3	0.0 (2)	C11—C6—C7—O2	−179.95 (18)
C4—N1—C2—O1	−11.0 (3)	C11—C6—C7—C8	−1.1 (3)
C4—N1—C2—C3	169.06 (17)	C5—C6—C11—C10	−175.71 (19)
C3—N2—C1—N1	0.3 (2)	C7—C6—C11—C10	1.1 (3)
C3—N2—C1—C13	179.15 (19)	O2—C7—C8—C9	179.22 (19)
C1—N2—C3—C2	−0.3 (2)	C6—C7—C8—C9	0.3 (3)
C1—N2—C3—C5	−179.1 (2)	C7—C8—C9—C10	0.7 (3)
N1—C1—C13—C14	−142.3 (2)	C8—C9—C10—C11	−0.7 (3)
N1—C1—C13—C18	41.4 (3)	C9—C10—C11—C6	−0.2 (3)
N2—C1—C13—C14	38.9 (3)	C1—C13—C14—C15	−177.4 (2)
N2—C1—C13—C18	−137.4 (2)	C18—C13—C14—C15	−1.1 (3)
O1—C2—C3—N2	−179.8 (2)	C1—C13—C18—C17	176.4 (2)
O1—C2—C3—C5	−0.8 (4)	C14—C13—C18—C17	0.1 (4)
N1—C2—C3—N2	0.2 (2)	C13—C14—C15—C16	1.0 (3)
N1—C2—C3—C5	179.09 (19)	C14—C15—C16—C17	0.0 (4)
N2—C3—C5—C6	3.6 (4)	C15—C16—C17—C18	−1.0 (4)
C2—C3—C5—C6	−175.1 (2)	C16—C17—C18—C13	0.9 (4)

### Hydrogen-bond geometry (Å, º)

**Table:**

*D*—H···*A*	*D*—H	H···*A*	*D*···*A*	*D*—H···*A*
O2—H2···N2	0.82	1.80	2.615 (2)	176
O4—H4···N4	0.82	1.79	2.612 (3)	175
C4—H4*A*···O1	0.96	2.56	2.896 (3)	100
C10—H10···O1^i^	0.93	2.58	3.403 (3)	148
C30—H30···O3^ii^	0.93	2.68	3.421 (3)	137
C24—H24*A*···O3	0.96	2.56	2.902 (3)	101

Symmetry codes: (i) −*x*+1, −*y*, −*z*; (ii) −*x*+1, −*y*+1, −*z*.
